# Comparative Study of Spheroids (3D) and Monolayer Cultures (2D) for the In Vitro Assessment of Cytotoxicity Induced by the Mycotoxins Sterigmatocystin, Ochratoxin A and Patulin

**DOI:** 10.3390/foods13040564

**Published:** 2024-02-13

**Authors:** Veronica Zingales, Maria Rosaria Esposito, Martina Quagliata, Elisa Cimetta, María-José Ruiz

**Affiliations:** 1 Research Group in Alternative Methods for Determining Toxics Effects and Risk Assessment of Contaminants and Mixtures (RiskTox), 46100 Valencia, Spain; m.jose.ruiz@uv.es; 2 Laboratory of Toxicology, Faculty of Pharmacy, University of Valencia, Av. Vicent Andrés Estellés s/n, 46100 Valencia, Spain; 3 Department of Industrial Engineering (DII), University of Padua, Via Marzolo 9, 35131 Padova, Italy; mresposito@rigenetics.com (M.R.E.); martina.quagliata@phd.unipd.it (M.Q.); elisa.cimetta@unipd.it (E.C.); 4 Fondazione Istituto di Ricerca Pediatrica Cittá Della Speranza (IRP)—Lab BIAMET, Corso Stati Uniti 4, 35127 Padova, Italy

**Keywords:** food contaminants, mycotoxins, cytotoxicity, 3D spheroid, tumour models, mesenchymal stem cells, endothelial cells, in vitro models

## Abstract

Mycotoxins are secondary metabolites produced by filamentous fungi associated with a variety of acute and chronic foodborne diseases. Current toxicology studies mainly rely on monolayer cell cultures and animal models, which are undeniably affected by several limitations. To bridge the gap between the current in vitro toxicology approach and the in vivo predictability of the data, we here investigated the cytotoxic effects induced by the mycotoxins sterigmatocystin (STE), ochratoxin A (OTA) and patulin (PAT) on different 2D and 3D cell cultures. We focused on human tumours (neuroblastoma SH-SY5Y cells and epithelial breast cancer MDA-MB-213 cells) and healthy cells (bone marrow-derived mesenchymal stem cells, BM-MSC, and umbilical vein endothelial cells, HUVECs). The cytotoxicity of STE, OTA, and PAT was determined after 24, 48 and 72 h of exposure using an ATP assay in both culture models. Three-dimensional spheroids’ morphology was also analysed using the MATLAB-based open source software AnaSP 1.4 version. Our results highlight how each cell line and different culture models showed specific sensitivities, reinforcing the importance of using more complex models for toxicology studies and a multiple cell line approach for an improved and more comprehensive risk assessment.

## 1. Introduction

Food contamination by toxic substances is a global safety concern, not only posing a serious threat to human and animal health but also having a massive economic impact on food industries [1]. Mycotoxins, natural toxic food and feed contaminants, are gaining increasing attention from the scientific community due to their abundance in food products and the harmful effects associated with their exposure. Mycotoxins are produced during the secondary metabolism of various fungal species under favourable climatic conditions of humidity and temperature [2]. They can build up in many types of food and feed crops in the field and during post-harvest activities such as improper handling procedures, packaging, storage, and transportation [3]. Human exposure may occur through the direct consumption of mycotoxin-contaminated plant-based food or from the consumption of products derived from animals fed with contaminated feed. Furthermore, mycotoxins are stable chemical compounds and persist even after food processing, cooking, baking, roasting, or pasteurization [4]. Their presence in food and feed has been associated with various pathological disorders in consumers, ranging from acute toxicity to cancer [5]. Due to their broad range of biological activities, mycotoxins represent a central issue in food safety worldwide. As a result, the Joint FAO/WHO Expert Committee on Food Additives (JECFA) has carried out risk assessments and set safety limits for many mycotoxins such as aflatoxins (AFs), deoxynivalenol, fumonisins, HT-2 and T-2 toxins, ochratoxin A (OTA), patulin (PAT) and zearalenone [6].

OTA and PAT are both mainly produced by *Aspergillus* spp. and *Penicillium* spp. fungi. While PAT is predominantly found in fruits and fruit-based products [7], OTA is considered one of the most prevalent contaminants in the food chain, occurring in cereals, grape, coffee, spices, and cocoa, as well as in foods of animal origin [8,9,10,11,12]. Epidemiological evidence suggests that OTA is implicated in the pathogenesis of renal diseases including Balkan endemic nephropathy, kidney tumours occurring in endemic regions of the Balkan Peninsula, and chronic interstitial nephropathy occurring in Northern African countries and likely in other parts of the world [13,14]. Hepatotoxic, teratogenic, neurotoxic, immunotoxic, genotoxic and carcinogenic effects have also been reported [15,16]. Despite evidence of its carcinogenicity in several animal studies, there is insufficient information to establish a causal link between OTA exposure and adverse effects in humans [17]. Accordingly, OTA has been classified as a possible human carcinogen (group 2B) by the International Agency for Research on Cancer (IARC) [18]. Since recent studies have raised uncertainty regarding the toxic properties of OTA, the European Food Safety Authority (EFSA) Panel on Contaminants in the Food Chain (CONTAM) established that the tolerable weekly intake (TWI) of 120 ng/kg body weight (bw) previously set in 2006 is no longer valid [19]. A recent OTA risk assessment set a new benchmark dose lower confidence limit for an extra cancer risk of 10% (BMDL_10_) at 4.73 µg/kg bw and 14.5 µg/kg bw as the non-neoplastic and neoplastic reference point, respectively [17].

PAT exposure has been related to neurotoxic, immunotoxic, embryotoxic and gastrointestinal effects, but the lack of strong evidence regarding its carcinogenicity led to its classification as a group 3 carcinogen (unclassifiable regarding its carcinogenicity in humans) by IARC [20,21]. Nevertheless, the adverse effects of PAT on human and animal health led regulatory agencies to establish a provisional maximum tolerable daily intake (PMTDI) of 0.4 µg/kg bw [22], and the maximum levels were set at ≤50 µg/kg in apple products and 10 µg/kg in baby food and infant formulae [23].

In addition to the regulated mycotoxins, there is a group of currently non-regulated ones for which no safety levels have been set due to the limited data regarding their occurrence and toxicity. The importance of establishing a better risk assessment for all of these contaminants is ever increasing, especially for sterigmatocystin (STE), a mycotoxin mainly produced by *Aspergillus* spp. and reported in grains and grain-based products, cheese, coffee, spices and beer [24,25,26,27,28,29]. STE is a biogenic precursor of the most potent carcinogenic known mycotoxin, AFB1, and shares several structural and biological properties with it, further highlighting the need for monitoring programs helping to define its maximum levels in food [30]. Animal studies have shown hepatotoxic and nephrotoxic effects induced by exposure to STE, and epidemiological evidence highlights a possible association between exposure to STE and an increased risk of developing tumours in humans [31,32,33,34,35]. As a result, IARC classified STE as a group 2B carcinogen [21].

Overall, there is a clear need to update the risk assessment for regulated and non-regulated mycotoxins, surpassing the limitations of current in vitro models. Risk assessment still mainly relies on cytotoxicity evaluations based on in vitro two-dimensional (2D) cell models, which undeniably provide poor predictions of in vivo conditions. Classical monolayer cultures lack both the complexity of a three-dimensional (3D) architecture and numerous biological factors that would allow them to reproduce cell and tissue physiology [36,37]. Scientific and technological advances can contribute to reducing the distance between the current in vitro toxicology and the true in vivo cell behaviour, thus increasing the reliability of the obtained data. In this context, the aim of the present study was to develop 3D cell culture models (spheroids) to assess the cytotoxic effects induced by individual exposure to the mycotoxins STE, OTA and PAT. For a more comprehensive risk assessment, we used different human tumour (neuroblastoma SH-SY5Y, and epithelial breast cancer MDA-MB-213 cells) and healthy cell lines (bone marrow-derived mesenchymal stem cells, BM-MSC, and umbilical vein endothelial cells, HUVECs). Several pieces of evidence led to the choice of these cell lines: (i) exposure to all three mycotoxins under study has been associated with neurotoxic effects [38,39,40]; however, the number of studies aimed at evaluating their effects on the neuronal system is limited; (ii) all three mycotoxins are known to gain entry into monogastric animals and humans in the intestinal tract, having epithelial cells as their primary target; however, the assessment of their effects on epithelial cancer cells, such as MDA-MB-231 cells, is very scarce; (iii) within the tumour models, one lineage of paediatric origin and one of adult origin were chosen, representing the wide spectrum of people who could be exposed to mycotoxins; and (iv) in vitro cytotoxicity tests are typically carried out in readily available and easily maintained immortalized cells, although they often do not reflect normal homologous cells, hence the decision to use MSCs, representing pre-tissue, multipotent cells found in different human tissues, and HUVECs, one of the most popular models used for endothelial cells in vitro [41,42]. In addition, evaluating the effects of mycotoxins on relevant cells lining human blood vessels can help us to understand the adverse effects caused by their contact with human blood vessels.

The results obtained for 3D spheroids were compared with those obtained in conventional 2D adherent cultures.

## 2. Materials and Methods

### 2.1. Reagents

The following reagent-grade chemicals and cell culture compounds were purchased from ATCC (American Type Culture Collection): DMEM high glucose culture medium with L-glutamine, foetal bovine serum (FBS), mesenchymal stem cell basal medium for adipose, umbilical and bone marrow-derived MSCs, the Mesenchymal Stem Cell Growth Kit for Bone Marrow-Derived MSCs, including recombinant human Fibroblast Growth Factor-basic (rh FGF-b), recombinant human insulin-like growth factor (rh IGF-1) and L-alanyl-L-glutamine. Endothelial cell growth medium, human epidermal growth factor (hEGF), vascular endothelial growth factor (VEGF), R3-insulin-like growth factor-1 (R3-IGF-1), ascorbic acid, hydrocortisone and human fibroblast growth factor-beta (hFGF-β) were obtained from PromoCell (Heidelberg, Germany). Minimum essential medium nonessential amino acids (MEM NEAA) and phosphate-buffered saline (PBS) were obtained from Gibco (Paisley, UK). Penicillin, streptomycin and trypsin/EDTA solutions were obtained from Corning (Rochester, NY, USA). The CellTiter-Glo 3D Cell Viability Assay was obtained from Promega (Madison, WI, USA). Standards of the selected mycotoxins, namely STE (MW: 324.28 g/mol), OTA (MW: 403.81 g/mol) and PAT (MW: 154.12 g/mol), as well as methanol (MeOH) were purchased from Sigma-Aldrich (St. Louis, MO, USA). Stock solutions of the mycotoxins were prepared in MeOH and maintained at −20 °C for STE and PAT and at +4 °C for OTA.

### 2.2. Cell Culture and Spheroid Formation

SH-SY5Y (ATCC CRL-2266) and MDA-MB-213 (ATCC HTB-26) cells were cultured in monolayer in DMEM high glucose with L-glutamine medium supplemented with 10% FBS, 1% MEM NEAA (100×) and 1% penicillin/streptomycin. BM-MSCs cells were cultured in Mesenchymal stem cell basal medium for adipose, umbilical and bone marrow-derived MSCs supplemented with 125 pg/mL rh FGF-b, 15 ng/mL rh IGF-1, 7% FBS, 2.4 mM L-alanyl-L-glutamine and 0.5% penicillin/streptomycin. HUVECs cells were cultured in endothelial cell growth medium supplemented with 0.5 mL of hEGF, 0.5 mL of VEGF, 0.5 mL of R3-IGF-1, 0.5 mL of ascorbic acid, 0.2 mL of hydrocortisone, 2 mL of hFGF-β and 2% FBS. BM-MSCs and HUVECs were used only up to passage 9. All cell lines were cultured under standard cell culture conditions at 37° C and 5% CO_2_ humidified atmosphere. The medium was changed every 2–3 days.

For spheroid generation, trypsinized single cells were suspended in the corresponding culture medium and dispensed into ultra-low attachment (ULA) 96-well round bottom plates (Corning^®^, New York, NY, USA). As previously described, the addition of 7.5 μg/mL collagen to the culture medium was required to form HUVEC spheroids [43]. For both tumour lines, cells were seeded at a density of 2 × 10^3^ cells/spheroid, while BM-MSCs and HUVECs were seeded at 5 × 10^3^ cells/spheroid and 7.5 × 10^3^ cells/spheroid, respectively. Plates were centrifuged at 1200 revolutions per minute (RPM) for 10 min to induce cell aggregation at the bottom of the wells. Prior to toxin exposure, BM-MSCs and HUVECs spheroids were cultured for 1 day, whereas MDA-MB-213 spheroids were cultured for 4 days and SH-SY5Y spheroids were cultured for 7 days, with a gentle 50% medium replenishment on day 4 for the latter. Seeding densities and growth times were optimized for all cell lines so that each spheroid exhibited appropriate diameter and shape parameters, according to Santo et al. [44] (Figure S1). Three-dimensional spheroids and monolayer cell cultures had the same culture media and growth conditions.

### 2.3. Treatment of Monolayer Cell Cultures and Spheroids

For monolayers, STE and OTA exposure was assessed in a concentration range of 1.56 to 50 µM for all cell lines. For PAT, the same concentrations were used for BM-MSCs and HUVECs, whereas concentrations from 0.035 to 1.12 µM and from 0.28 to 9 µM were employed for SH-SY5Y and MDA-MB-231 cells, respectively. The concentration range for STE and OTA was established considering their occurrence in food and the half maximal inhibitory concentrations (IC_50_) available from the literature [30,45]. Lower concentrations were selected for PAT based on our pilot studies.

Considering the routinely lower sensitivity of 3D cultures [46,47], spheroids were exposed to higher mycotoxin concentrations. STE and OTA were used in a range from 6.25 to 100 µM for all cell lines. For PAT, the same concentrations were used for BM-MSCs and HUVECs spheroids, whereas concentrations between 3.12 and 12.5 µM and between 6.25 and 25 µM were employed for SH-SY5Y and MDA-MB-231 spheroids, respectively. Five to six serial concentrations (dilution factor = 2) of each mycotoxin were tested in both cell culture systems. The range of mycotoxins concentrations used in the study are summarized in Table S1.

Cells and spheroids were exposed to the individual mycotoxins for 24, 48 and 72 h. During the exposure time, neither the medium nor the mycotoxins were replenished. All mycotoxins were diluted to the desired concentration in the appropriate culture medium used for cell growth. Solvent controls containing the same amount of MeOH were included in each experiment.

### 2.4. Morphological Analysis

Bright-field images of spheroids at time 0 and after 72 h of exposure to the mycotoxins were obtained using an inverted light microscope Zeiss Primo Vert equipped with a Zeiss camera (Axiocam 208 color, Zeiss Microscopy, Berlin, Germany). All images were analysed using the open source software AnaSP 1.4 version run with MATLAB R2022a (Version 9.12) and Image Processing Toolbox. The following parameters were extracted: circularity, compactness, solidity, area, and volume. Circularity (*Cir*) is a measure of how close the shape of the spheroid image is to a circle. It was used to calculate the Sphericity Index (SI), according to Equation (1):

(1) $$\mathrm{SI}=\sqrt{Cir}$$

The parameter “compactness” is measured by dividing spheroid area by its squared perimeter, with the circle being the object with the most compact shape and, therefore, with the maximum value of compactness, which is 1 [48]. Finally, solidity, which is an indicator of the roughness of the spheroidal surface, was determined in order to assess spheroids’ regularity [49].

### 2.5. Cell Viability Assay

The CellTiter-Glo 3D Cell Viability Assay (Promega^®^, G968B, Madison, WI, USA) was performed to evaluate the effects on cell viability induced by STE, OTA, and PAT exposure in both cell modalities and according to the manufacturer’s instructions. This assay is based on the properties of a thermostable luciferase, which generates a luminescent signal proportional to the amount of adenosine triphosphate (ATP) present, a marker for metabolically active cells. Briefly, 100 µL/well of the culture medium was replaced with a medium containing mycotoxins at the desired concentrations (see Section 2.3 for the detailed concentrations tested). After the exposure time, 100 µL/well of the medium was replaced with an equal volume of CellTiter-Glo Reagent. Plates were gently shaken for 5 min to induce cell lysis and then incubated at room temperature for an additional 25 min to stabilize the luminescent signal, keeping the plates in the dark. After the incubation time, 100 µL of the solution from each well was transferred to an opaque-walled flat-bottom multiwell plate. Luminescence was recorded at 570 nm using Spark^®^ Multimode Microplate Reader by Tecan (Männedorf, Switzerland).

A similar protocol was used for monolayers: 100 μL trypsinized cell suspensions were seeded in 96-well flat-bottom plates at a density of 7.5 × 10^3^ cells/well for HUVECs and SH-SY5Y cells, and 5 × 10^3^ cells/well for BM-MSCs and MDA-MB-231. The plates were then incubated and when cells reached 80% confluence, a fresh medium containing the mycotoxins at the desired concentrations was added (see Section 2.3 for the detailed concentrations tested). After the exposure time, the spent culture medium was removed and replaced with 50 µL/well of fresh medium and an equal volume of CellTiter-Glo^®^ Reagent (Promega). The incubation time was reduced to 2 min under shaking and to 10 min at room temperature under static conditions. The luminescence signal was recorded on 100 µL of solution transferred from each well to an opaque flat-bottom multiwell plate, as reported above. Three independent experiments for each condition were carried out. For both cell culture systems, cell viability was expressed as a percentage relative to the solvent control (MeOH). The IC_50_ values were calculated using Graphpad Prism version 8.0.2 (nonlinear regression (curve fit) [Inhibitor] vs. normalized response; GraphPad Software, San Diego, CA, USA).

### 2.6. Estimation of LD_50_ Based on In Vitro IC_50_ Value

To prove the accuracy of the results obtained in 3D cultures, the formula developed by the Interagency Coordinating Committee on the Validation of Alternative Methods (ICCVAM) was applied [50]. According to this, the dose that produces lethality in 50% of the animals tested (LD_50_) of acute oral toxicity was estimated from in vitro IC_50_ by using Equation (2):

log LD_50_ = 0.372 × log IC_50_ (µg/mL) + 2.024 (2)

### 2.7. Statistical Analysis

Statistical analysis was carried out using GraphPad Prism version 8.0.2 (GraphPad Software, San Diego, CA, USA), statistical software package. Data were expressed as mean ± SEM of different independent experiments. We also performed Student’s *t*-test for paired samples and the differences between groups were assessed via two-way ANOVA followed by the Tukey HDS post hoc test for multiple comparisons. We set a significance level of 0.05, such that *p* ≤ 0.05 was considered statistically significant.

## 3. Results

### 3.1. Cytotoxic Effects of STE, OTA and PAT in Monolayer Cultures

**Figure 1:**
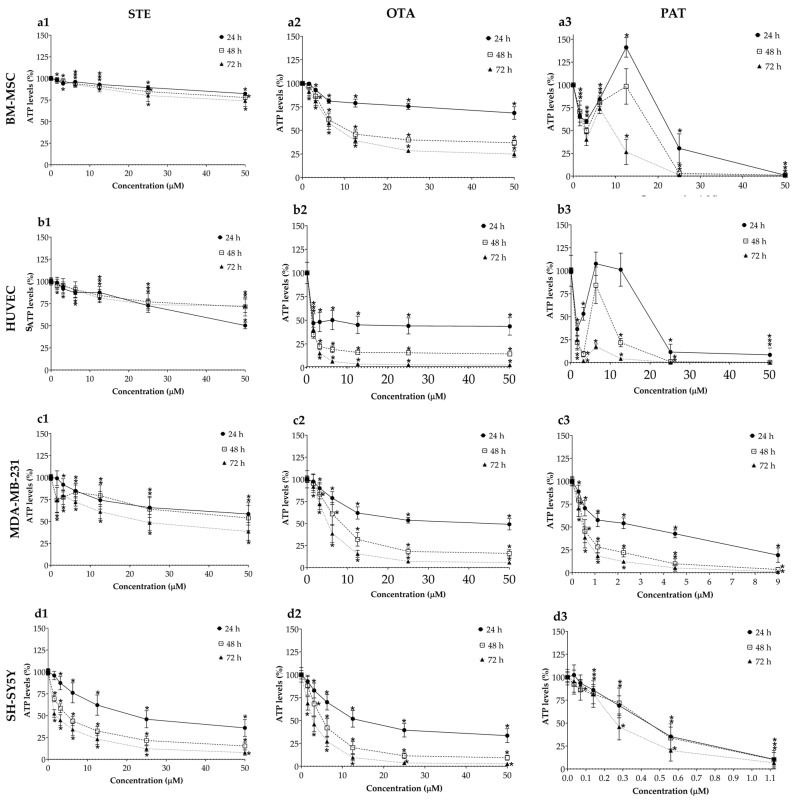
Cytotoxic effects of STE (column 1, **a1**,**b1**,**c1**,**d1**), OTA (column 2, **a2**,**b2**,**c2**,**d2**) and PAT (column 3, **a3**,**b3**,**c3**,**d3**) on BM-MSCs (row **a**), HUVECs (row **b**), MDA-MB-231 (row **c**) and SH-SY5Y (row **d**) cells cultured in monolayer. Cell viability was determined by means of the ATP assay after 24, 48 and 72 h of exposure. Data are expressed as the mean ± SEM of three independent experiments (*n* = 3). (*) *p* ≤ 0.05 indicates a significant difference compared to the control.

The cytotoxic effects of STE, OTA and PAT were assessed by means of the ATP assay after 24, 48 and 72 h of exposure for all cell lines cultured in 2D monolayers and the resulting concentration–response curves are shown in Figure 1. STE (column 1 in Figure 1) had a significant and stronger effect on cancer cell cultures even at the lowest concentrations tested and resulted only in moderate viability reductions for the healthy BM-MSCs and HUVECs lines, with a reduction in cell viability ranging from 2% to 26% and from 0.3% to 50%, respectively. OTA (column 2 in Figure 1) was the mycotoxin which most consistently induced concentration- and time-dependent reductions in viability across all cell lines, both cancerous and healthy, with rapid drops in HUVECs’ viability even at the lowest doses. Measurements following PAT exposure (column 3 in Figure 1) resulted in more complex patterns of ATP level fluctuations, which led to the highest cell death percentages (up to 99%, 92%, 81% and 90% in BM-MSCs, HUVECs, MDA-MB-231 and SH-SY5Y cells, respectively) even at the shortest 24 h exposure time. Interestingly, the toxic impacts of 72 h of exposure to PAT were similar in HUVECs, MDA-MB-231 and SH-SY5Y cells, with IC_50_ values equal to 0.45 ± 0.18 µM, 0.42 ± 0.13 µM and 0.28 ± 0.15 µM, respectively. Table 1 summarizes all calculated IC_50_ values obtained by means of the ATP assay on BM-MSCs, HUVECs, MDA-MB-231 and SH-SY5Y cells after exposure to STE, OTA and PAT at the three different time points.

### 3.2. Cytotoxic Effects of STE, OTA and PAT in 3D Spheroids

**Figure 2:**
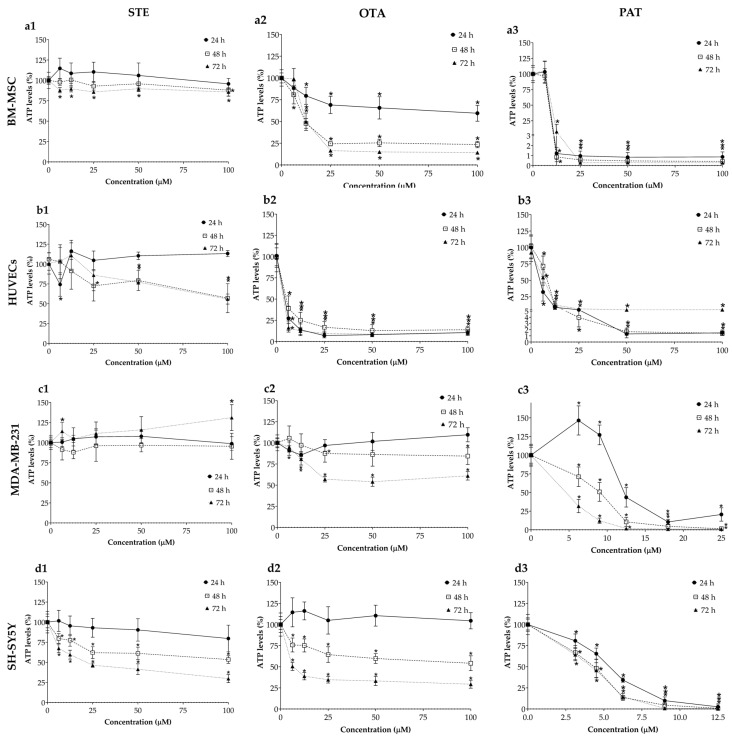
Cytotoxic effects of STE (column 1, **a1**,**b1**,**c1**), OTA (column 2, **a2**,**b2**,**c2**,**d2**) and PAT (column 3, **a3**,**b3**,**c3**,**d3**) on BM-MSCs (row **a**), HUVECs (row **b**), MDA-MB-231 (row **c**) and SH-SY5Y (row **d**) spheroids. Cell viability was determined by means of the ATP assay after 24, 48 and 72 h of exposure. Data are expressed as the mean ± SEM of three independent experiments (*n* = 3). (*) *p* ≤ 0.05 indicates a significant difference compared to the control.

The cytotoxic effects of STE, OTA and PAT were also investigated in BM-MSCs, HUVECs, MDA-MB-231 and SH-SY5Y spheroids by means of the ATP assay after 24, 48 and 72 h of exposure (Figure 2). As expected, the responses measured in our 3D models differ from those obtained in conventional 2D monolayer systems. While STE exposure (column 1 in Figure 2) still significantly affected the viability of SH-SY5Y spheroids, MDA-MB-231 cells, the other tumour line we used, did not show relevant changes in ATP levels. Regarding spheroids derived from healthy cells, a significant decrease in cell viability was also obtained in BM-MSC spheroids after 72 h of exposure, as well as in HUVEC spheroids exposed to the concentrations 25–100 µM for 48 and 72 h. Interestingly, in HUVEC spheroids exposed to STE for 24 h, a significant reduction in cell viability was induced only by the lowest concentration (6.25 µM). OTA (column 2 in Figure 2) again had potent and rapid toxic effects on both BM-MSCs and HUVECs, while spheroids formed from the two cancer cell lines maintained higher viabilities among all the concentrations and exposure times tested. Finally, significant cytotoxicity was induced by almost all tested concentrations of PAT (column 3 in Figure 2) in each cell line starting from 24 h of exposure. Noteworthily, the toxic effect of PAT seems to be unrelated to the time of exposure, as no relevant differences can be noticed between the different times tested. Table 1 summarizes the IC_50_ values obtained by ATP assay on BM-MSCs, HUVECs, MDA-MB-231 and SH-SY5Y cells after exposure to STE, OTA and PAT at the three different time points.

### 3.3. Analysis of Morphological Parameters in 3D Cultures Exposed to STE, OTA and PAT

**Figure 3:**
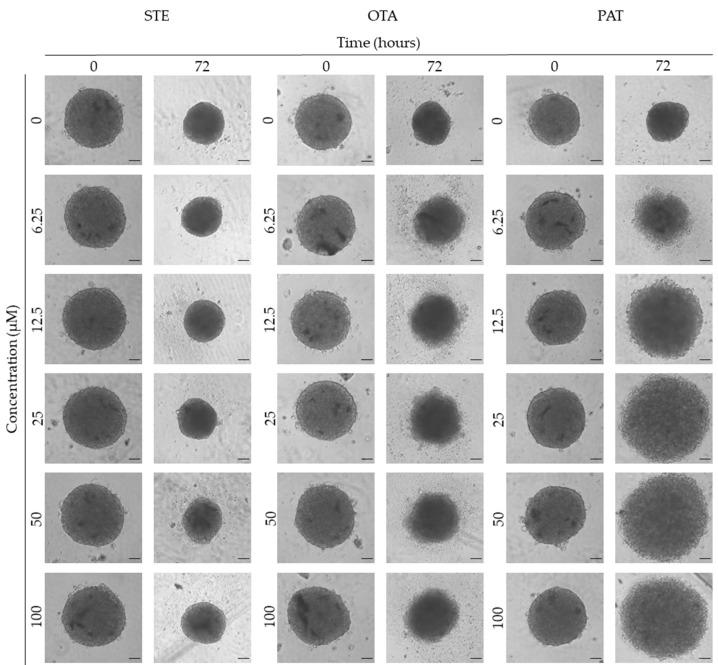
Representative bright-field images of BM-MSC spheroids after 0 and 72 h of exposure to STE, OTA and PAT. Spheroids exposed to the same amount of solvent (MeOH) were used as the control. Images were obtained using the Light Microscope Zeiss Axio Observer (Zeiss Microscopy, Germany). Scale bar: 100 μm (magnification 5×).

**Figure 4:**
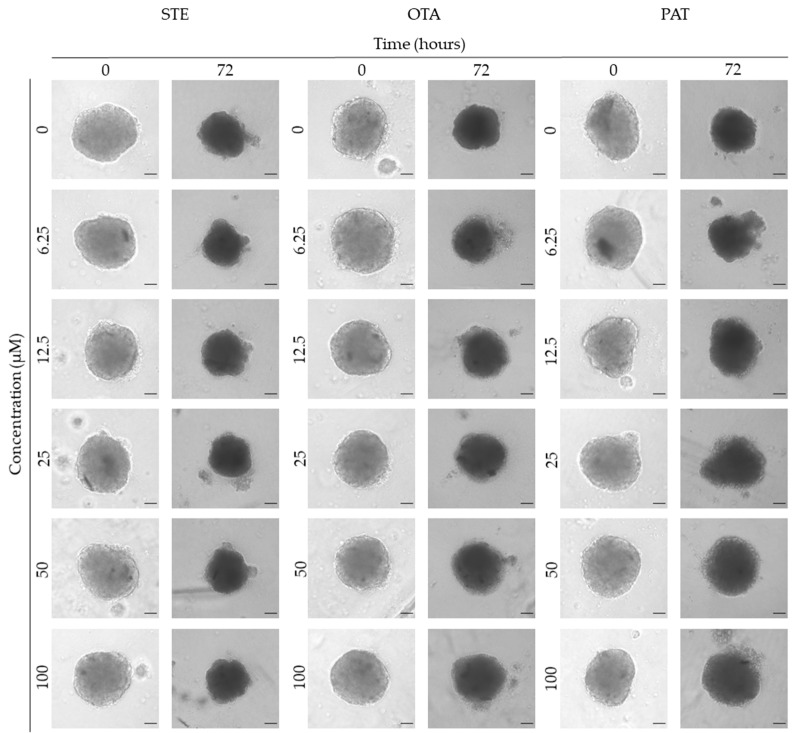
Representative bright-field images of HUVEC spheroids after 0 and 72 h of exposure to STE, OTA and PAT. Spheroids exposed to the same amount of solvent (MeOH) were used as the control. Images were obtained using the Light Microscope Zeiss Axio Observer (Zeiss Microscopy, Germany). Scale bar: 100 μm (magnification 5×).

**Figure 5:**
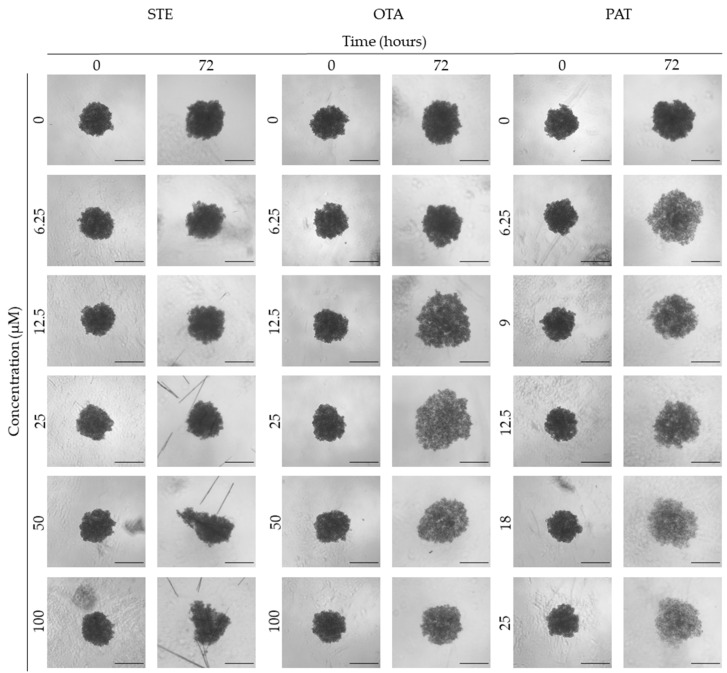
Representative bright-field images of MDA-MB-231 spheroids after 0 and 72 h of exposure to STE, OTA and PAT. Spheroids exposed to the same amount of solvent (MeOH) were used as the control. Images were obtained using the Light Microscope Zeiss Axio Observer (Zeiss Microscopy, Germany). Scale bar: 500 μm (magnification 2×).

**Figure 6:**
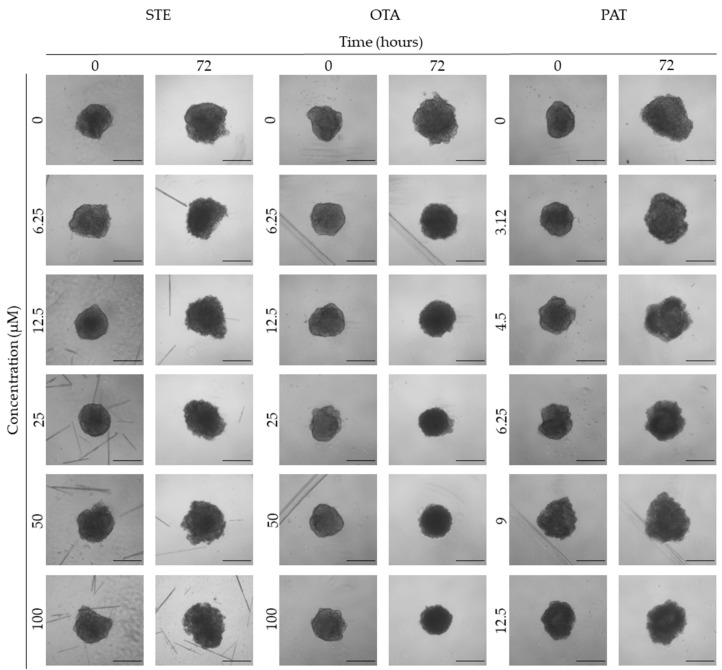
Representative bright-field images of SH-SY5Y spheroids after 0 and 72 h of exposure to STE, OTA and PAT. Spheroids exposed to the same amount of solvent (MeOH) were used as the control. Images were obtained using the Light Microscope Zeiss Axio Observer (Zeiss Microscopy, Germany). Scale bar: 500 μm (magnification 2×).

**Figure 7:**
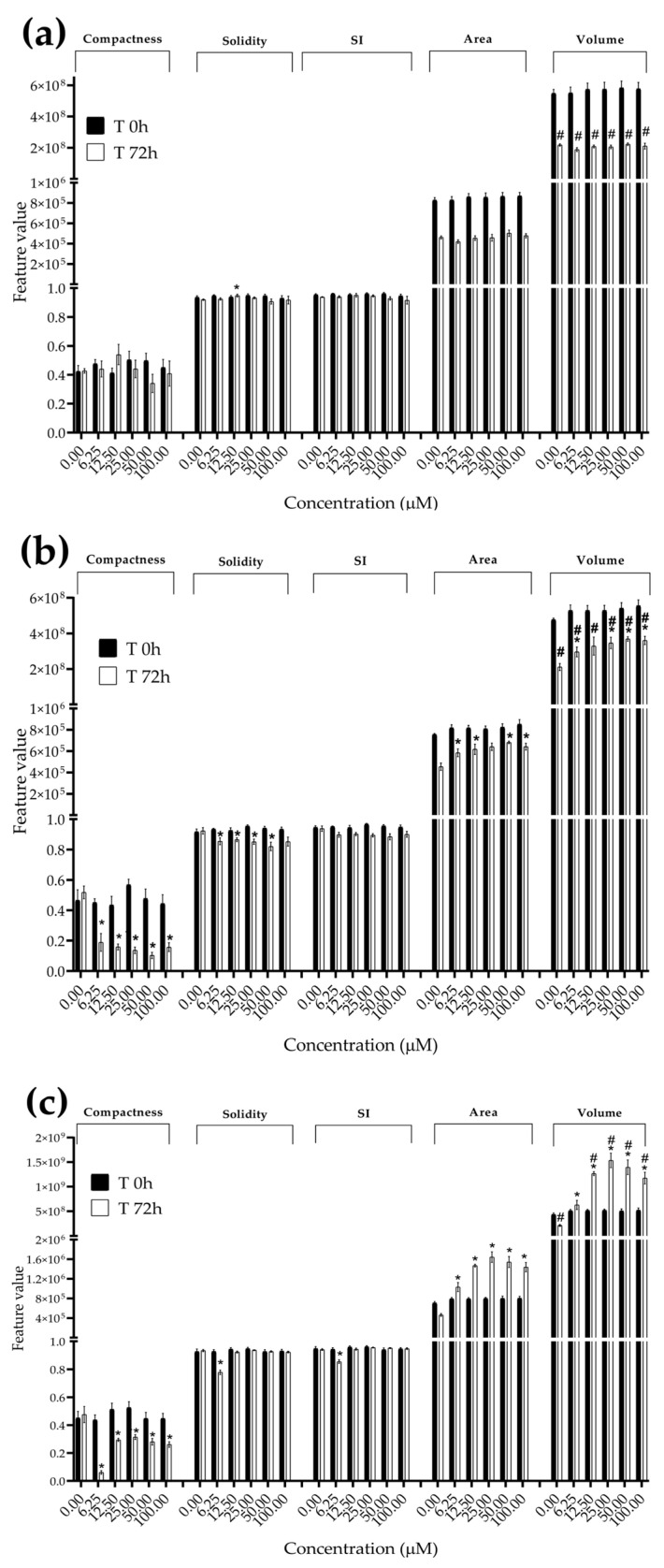
Morphological parameters of BM-MSC spheroids after 0 and 72 h of exposure to (**a**) STE, (**b**) OTA and (**c**) PAT. Compactness, solidity, SI, area and volume were determined after 0 and 72 h of exposure using AnaSP software. Data are expressed as the mean ± SEM of three independent experiments (*n* = 3). (*) *p* ≤ 0.05 indicates a significant difference compared to the control. (#) *p* ≤ 0.05 indicates a significant difference compared to the respective T 0 h.

**Figure 8:**
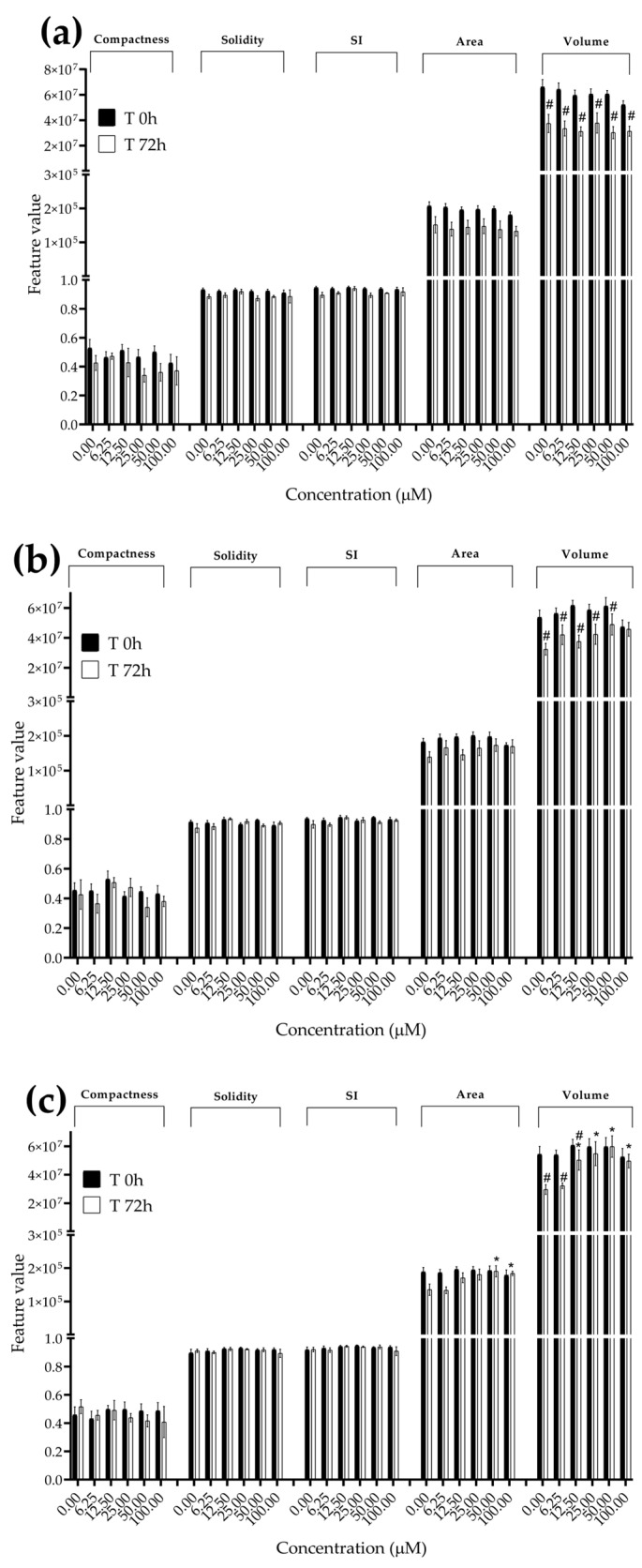
Morphological parameters of HUVEC spheroids after 0 and 72 h of exposure to (**a**) STE, (**b**) OTA and (**c**) PAT. Compactness, solidity, SI, area and volume were determined after 0 and 72 h of exposure using AnaSP software. Data are expressed as the mean ± SEM of three independent experiments (*n* = 3). (*) *p* ≤ 0.05 indicates a significant difference compared to the control. (#) *p* ≤ 0.05 indicates a significant difference compared to the respective T 0 h.

**Figure 9:**
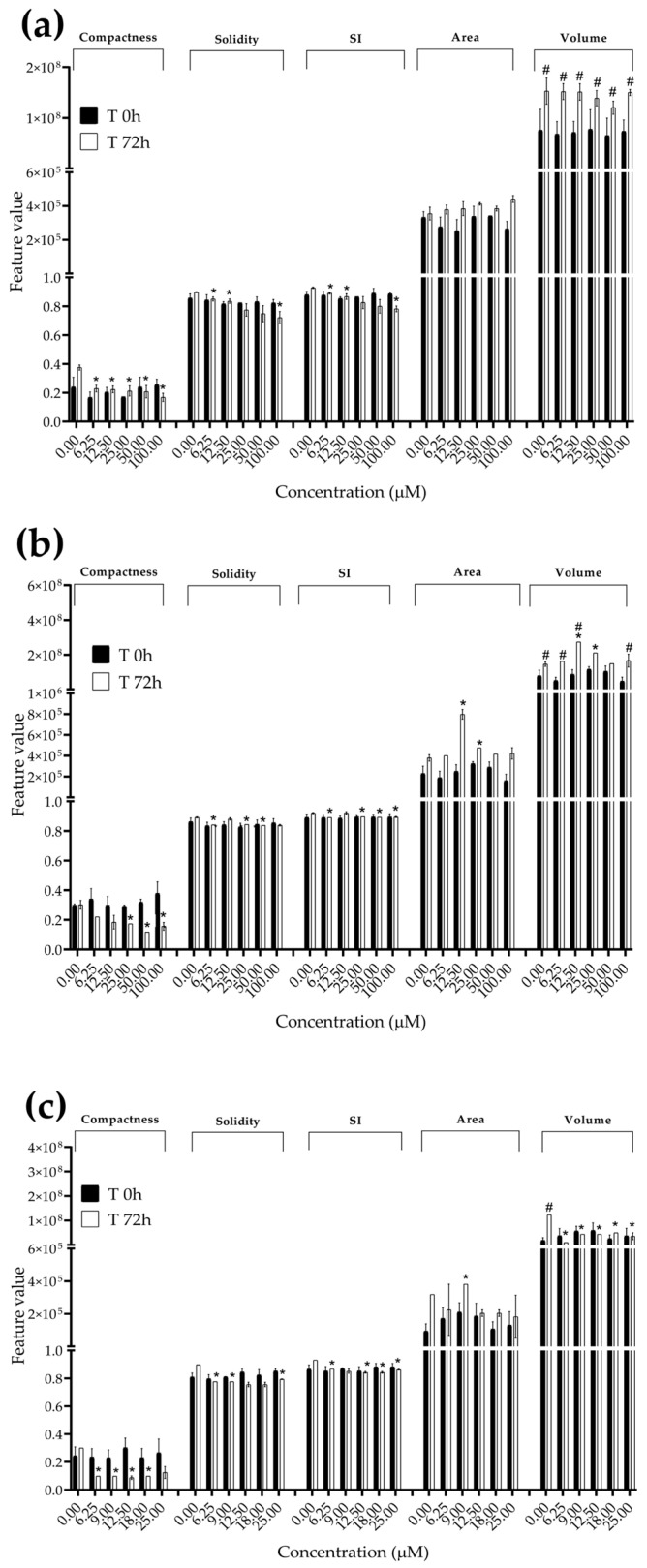
Morphological parameters of MDA-MB-231 spheroids after 0 and 72 h of exposure to (**a**) STE, (**b**) OTA and (**c**) PAT. Compactness, solidity, SI, area and volume were determined after 0 and 72 h of exposure using AnaSP software. Data are expressed as the mean ± SEM of three independent experiments (*n* = 3). (*) *p* ≤ 0.05 indicates a significant difference compared to the control. (#) *p* ≤ 0.05 indicates a significant difference compared to the respective T 0 h.

**Figure 10:**
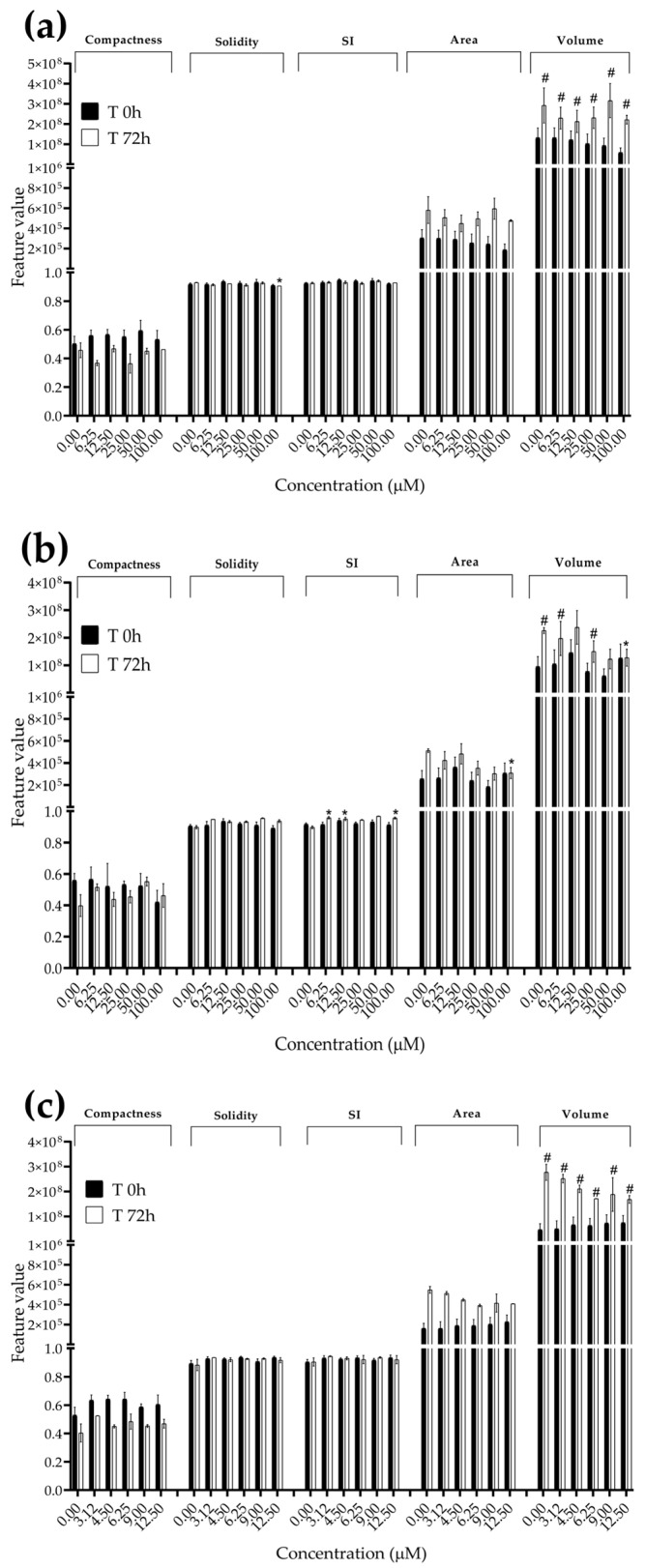
Morphological parameters of SH-SY5Y spheroids after 0 and 72 h of exposure to (**a**) STE, (**b**) OTA and (**c**) PAT. Compactness, solidity, SI, area and volume were determined after 0 and 72 h of exposure using AnaSP software. Data are expressed as the mean ± SEM of three independent experiments (*n* = 3). (*) *p* ≤ 0.05 indicates a significant difference compared to the control. (#) *p* ≤ 0.05 indicates a significant difference compared to the respective T 0 h.

We next investigated the effects of mycotoxin treatment on spheroids’ organization and morphology. To achieve this, we captured brightfield images of the spheroids before and after 72 h of mycotoxin exposure and performed 3D image analysis (Figure 3, Figure 4, Figure 5, Figure 6, Figure 7, Figure 8, Figure 9 and Figure 10). Noteworthy, the quantitative analysis performed using AnaSP software revealed different behaviours in tumour and non-tumour spheroids over time. In particular, untreated tumour spheroids increase in size over 72 h, while those derived from the healthy BM-MSCs and HUVEC cells undergo a significant decrease in volume and an increase in compactness. SI did not vary significantly over time for all cell lines, and solidity was comparable in spheroids at T 0 h and T 72 h (Figure 7, Figure 8, Figure 9 and Figure 10).

Following treatment, BM-MSC spheroids exposed to OTA and PAT lost compactness and acquired a frayed and uneven surface appearance, in contrast to the compact smooth structure of the controls (Figure 3). The reduction in compactness led to an increase in spheroid size (area and volume), in particular in BM-MSC spheroids exposed to the highest concentrations of PAT. Automated evaluation of the morphological parameters confirmed this visual observation (Figure 7b,c). As expected, no significant morphological alterations were observed in BM-MSC spheroids exposed to STE for 72 h compared to the control (Figure 3 and Figure 7a). Visually, HUVEC spheroids present a slight disruption on the outer layers and an accumulation of cell debris after exposure to OTA and PAT (Figure 4). These observations were, however, not confirmed by image analysis, which revealed only a significant increase in size after exposure to the highest concentrations of PAT (Figure 8c). Conversely, tumour MDA-MB-231 spheroids exposed to each of the three mycotoxins appeared visibly disaggregated, with cells detaching from the structure (Figure 5). These observations were confirmed by measurements of significant decreases in compactness, solidity and SI at most of the concentrations tested (Figure 9). Finally, images from neuroblastoma SH-SY5Y spheroids are presented in Figure 6 and quantified in Figure 10. Mycotoxin treatment led to evident morphological alterations, but only 100 µM STE and 100 OTA µM induced significant decreases in solidity and size (area and volume) compared to the control, respectively (Figure 10a,b).

### 3.4. LD_50_ Estimation for In Vivo Study Based on In Vitro IC_50_ Data

According to ICCVAM [50], the LD_50_ value of rats can be estimated from the IC_50_ value obtained from the in vitro cytotoxicity assay by using the regression formula described previously (see Section 2.6). Using this approach, a predictive model was built to find out whether the in vivo LD_50_ values of rats could be estimated from the IC_50_ values obtained in spheroids from the in vitro cytotoxic ATP assay. To determine the estimated range of LD_50_ values, the highest and lowest IC_50_ values obtained for each mycotoxin were selected for each cell line. For those conditions for which no IC_50_ value was determined in the range of concentrations assayed, the maximum concentration tested was considered. Based on the results, the LD_50_ values ranged between 385.53 and 287.49 ± 98.04 mg/kg for STE, between 380.95 ± 37.36 and 119.9 ± 18.77 mg/kg for OTA and from 125.56 ± 48.98 to 86.20 ± 9.21 mg/kg for PAT (Table 2).

## 4. Discussion

Although great strides have been made in the risk assessment of mycotoxins through conventional toxicity testing and animal bioassays, the challenge remains for regulators to establish even more accurate safety levels for many of these natural contaminants. The need for a more comprehensive evaluation of the effects of STE, OTA and PAT mycotoxins is based on their high occurrence and/or noteworthy toxicity. As a matter of fact, in recent years, many researchers have provided cytotoxicity data concerning OTA and PAT exposure, while there is an increasing awareness of the importance of establishing a better risk assessment for STE [30,45]. Interestingly, the evaluation of the effects of these mycotoxins has mainly been carried out on monolayer cells, which are poor indicators of the real human hazard. The modernization of food toxicology via the integration of technological advances is endorsed by the Food and Drug Agency (FDA) and the EFSA, with emphasis on prioritizing advanced non-animal testing models for safety testing [51,52]. Accordingly, the new era of food toxicology is relying more and more on biorelevant in vitro systems [43]. Nonetheless, evaluations of STE, OTA and PAT toxicity using new alternative methods are still scarce [53,54,55]. In this context, the present study explored the cytotoxicity of the mycotoxins STE, OTA and PAT using two different culture models, traditional 2D cultures and 3D spheroids. Moreover, for a more comprehensive evaluation, we used a panel of cell lines which included tumour and non-tumour samples. Our results reveal a wide variability in STE, OTA and PAT cytotoxicity among the different cell lines tested. Our findings agree with the literature, in which different cells have been shown to have a different sensitivity to mycotoxin exposure. In particular, IC_50_ values ranging from 1.86 µM to >200 µM were reported for OTA in monolayer cells, depending on the experimental conditions, the time of exposure and the cell line used. Even more variability was obtained for PAT, with IC_50_ values varying from µM to mM [45]. Regarding STE, few studies have investigated its cytotoxicity so far. However, based on the current literature, great variability has also been demonstrated in its cytotoxic effects, with IC_50_ values ranging between 3.7 µM and 286.1 µM [30]. Considering our results for monolayer cells, SH-SY5Y cells were the most sensitive to STE and PAT, with IC_50_ values ranging from 28.22 ± 11 µM to 2.91 ± 1.04 µM and from 0.45 ± 0.16 µM to 0.28 ± 0.15 µM, respectively. Regarding OTA, the lowest IC_50_ values were obtained in HUVECs, with values ranging from 13.87 ± 6.40 µM to 0.80 ± 0.06 µM. Similar results were obtained in 3D models, with SH-SY5Y spheroids being the most sensitive to STE and PAT and HUVEC spheroids being the most sensitive to OTA. However, differences of up to one order of magnitude in IC_50_ values can be observed between the two culture models for almost all conditions tested. Based on literature evidence, spheroids are expected to show a considerable increased resistance to toxic substances compared to 2D cells due to the pronounced intracellular junctions and a dense extracellular matrix with small pores, which influence xenobiotic transport by decreasing its penetration [46,47]. Therefore, the absence of a 3D organization in 2D systems can lead to an overestimation of cellular toxicity. Interestingly, while this agrees with what we observed in tumour cell lines, the same cannot be said for healthy non-tumour cells. In fact, while on the one hand SH-SY5Y and MDA-MB-231 spheroids showed higher IC_50_ values compared to monolayer cells, on the other hand, quite similar results were obtained in BM-MSCs cultured as monolayer cells and spheroids, while HUVEC spheroids showed a lower resistance compared to monolayer cells in almost all conditions assayed, with the exception of 48 and 72 h of PAT exposure. Similarly, Kim et al. showed that hepatospheroids were more sensitive to fumonisin B1 than monolayer-cultured hepatocytes [56], confirming that, in some cases, the evaluation of toxicity on monolayer cultures may lead to an underestimation of the effects.

From the three tested mycotoxins, PAT caused the highest toxicity in all cell models. Many animal-based studies have demonstrated the high toxic properties of PAT. However, the lack of strong evidence of its carcinogenicity has prompted its classification as a group 3 carcinogen by IARC [20,21]. Interestingly, in our study, the toxic impact of OTA (a group 2B carcinogen) and PAT was similar in HUVECs. Finally, it should be noted that in cells cultured as spheroids, which better represent the in vivo cell behaviour, mycotoxins greatly affected non-tumour cell lines, suggesting that an evaluation of the effects of mycotoxins on cancer cells may lead to an underestimation of their toxicity, due to the intrinsic ability of tumour cells to survive at relevant concentrations of a toxic compound [57].

The simplicity and homogeneity that characterize 2D cultures are reflected in the linear time- and concentration-dependent decrease in cell viability following exposure to STE, OTA and PAT (Figure 1). Conversely, spheroids possess an inherent complexity that represents a significant challenge in spheroid-based assays for toxicity testing [58]. To reduce variations between samples and within repeats, an accurate spheroid generation method was followed, as previously described [43]. Noteworthy, a less linear trend in cell viability decrease was observed in 2D BM-MSCs and HUVECs, as well as in MDA-MB-231 spheroids exposed to PAT. Based on our results, it could be assumed that in these cell cultures PAT induced a hormetic response, characterized by stimulation at low concentrations and inhibition at high concentrations [59]. Moreover, while in HUVECs, a restoration of vitality to control levels can be observed at 24 h of exposure at the intermediate concentrations (6.25 and 12.5 µM), in 2D BM-MSCs and MDA-MB-231 spheroids a significant increase up to 141% and 146%, respectively, was measured. To explain these data, it should be considered that the assay measures the ATP molecules released by cells. While, on the one hand, ATP is a marker for the presence of metabolically active cells, on the other, molecular mechanisms including programmed cell death and autophagy require the production of a lot of energy and are positively correlated with cellular ATP levels [60]. Therefore, an increase in the percentage of ATP may also be due to the establishment of cellular processes that allow them to recover the biological material of the dying cells and isolate the damage. In support of this, MDA-MB-231 spheroids exposed to PAT 6.25 and 9 µM showed an increased number of dead cells compared to the control, while an altered morphology was observed in 2D BM-MSCs exposed to PAT 12.5 µM (Figures S2 and S3). Furthermore, it has been demonstrated that PAT is able to induce ROS-dependent autophagic cell death in human hepatoma G2 (HepG2) cells [61]. However, further investigations are required to better understand whether autophagy or other molecular mechanisms underlie the increased ATP levels that we observed.

The morphological analysis of spheroids is emerging as one of the most reliable and cost-effective ways to determine the cytotoxic effects of a treatment with minimal experimental manipulation [62]. Similarly to Mittler et al. and Aguilar Cosme et al. [58,63], sphericity was found to be rather independent from spheroid damage after treatment. On the contrary, compactness and size seem to be the main parameters corroborating the results of the viability assays. Although a reduction in spheroid volume and area is one of the most striking features in response to cytotoxic compounds [62], in our study, the cytotoxic effects induced by mycotoxin exposure were related to an increase in size of non-tumour spheroids. In particular, marked morphological alterations were noted in BM-MSC spheroids exposed to the highest concentrations of PAT, in which a larger and loosened appearance was acquired. This change can be attributed to a reduction in cell–cell interactions and the loss of adherens junctions (Figure S4), as previously reported by Celli et al. [64]. However, according to our findings, the image-based analysis results were limited in most of the conditions assayed, not showing significant morphological alterations that could be presumed from visual impressions. This may be partly due to limitations arising from working with low-resolution bright-field microscopy images. On the other hand, it should be taken into account that the morphological disruption is cell-type-dependent and that the toxic response is heterogeneous and not always limited to morphological changes [65,66].

Traditionally, the safety of a substance is determined through in vivo acute oral toxicity test by estimating its LD_50_ value. According to the ICCVAM [50], the LD_50_ value of rats can be estimated from the IC_50_ value obtained from in vitro cytotoxicity assays, reducing the number of animals needed for range finding. Previous in vivo studies determined the LD_50_ of STE in rats to be in the range of 60–85 mg/kg bw [30], while LD_50_ values equal to 20–30.3 mg/kg bw and in the range of 20–100 mg/kg bw were found in rats exposed to OTA and PAT, respectively [17,67]. Interestingly, in almost all cell lines, the calculated LD_50_ of PAT falls within the range of the in vivo toxicity established for rats, confirming that an in vitro cytotoxicity test performed in spheroids may predict in vivo toxicity. However, for STE and OTA, the LD_50_ values estimated using the ICCVAM approach were higher than those obtained in animal studies. This could be due to the differences between cell cultures and whole animals in regard to the absorption, distribution, availability, metabolism and excretion of tested compounds, which result in a level of accuracy of this method in predicting the LD_50_ value of around 30%, as reported for other substances tested in the validation study [50]. Furthermore, it should be noted that we used cells of human origin, and thus the comparison between the calculated LD_50_ values and studies performed in rodents may lead to some uncertainties, as observed in previous studies [68,69].

In conclusion, our study confirms the importance of performing mycotoxin cytotoxicity screening in more complex and in vivo-like culture models, as the effects found in monolayers were not reproducible in 3D, as well as differing between cell lines. The SH-SY5Y cell line was the most sensitive to STE and PAT and HUVECs were the most sensitive to OTA. Meanwhile, comparing the three mycotoxins, PAT caused the highest cytotoxicity. However, a different behaviour was observed between tumour and healthy cell lines, with the former more resistant to mycotoxins when cultured as spheroids, and the latter when cultured as monolayers. Furthermore, when comparing tumour and non-tumour cell lines, a stronger cytotoxic effect can be observed in the latter, which suggests that an evaluation of the effects of mycotoxins on cancer cells may lead to an underestimation of their toxicity. Finally, based on our data, the results from 3D cultures partly confirm previous studies performed on animal models, although a complete translation of the results from human spheroids to animal shows evident limitations. Overall, our data stress the evidence that the toxicity of mycotoxins cannot be predicted solely based on the effects on a single cell model. In this context, the present study is intended to be the springboard from which future research directions may also be highlighted. In this sense, the importance of evaluating the effects induced by mycotoxin mixtures through a new toxicological approach based on the use of advanced alternative methods is noteworthy. Evidence indicates that consumers are likely exposed to more than one mycotoxin at the same time, producing a new toxicity scenario that may be different than expected. The implementation of different predictive advanced models for individual and combined mycotoxin toxicity is a valuable tool to estimate the true risk of harmfulness and establish regulatory standards in food and feed. The findings and their implications should be discussed in the broadest context possible and interpreted from the perspective of previous studies and of the working hypotheses.

## Figures and Tables

**Table 1:** The half maximum inhibitory concentration (IC_50_) values of STE, OTA and PAT in BM-MSCs, HUVECs, MDA-MB-231 and SH-SY5Y cultured as monolayers and spheroids. Data are expressed as the mean ± SEM of three independent experiments (*n* = 3).

IC_50_ Value (µM) ± SEM							
		2D			3D		
Cell Line	Mycotoxin	Time of Exposure			Time of Exposure		
		24 h	48 h	72 h	24 h	48 h	72 h
BM-MSCs	STE	>50 *	>50 *	>50 *	>100 *	>100 *	>100 *
	OTA	>50 *	14.31 ± 2.36	10.43 ± 1.35	>100 *	15.80 ± 2.02	17.11 ± 2.38
	PAT	29.09 ± 7.28	13.38 ± 3.62	5.43 ± 1.86	9.52 ± 1.28	8.40 ± 1.01	9.98 ± 1.88
HUVECs	STE	>50 *	>50 *	>50 *	>100 *	>100 *	>100 *
	OTA	13.87 ± 6.40	1.09 ± 0.24	0.80 ± 0.06	2.59 ± 1.38	5.14 ± 2.06	2.20 ± 0.71
	PAT	19.99 ± 7.44	1.49 ± 0.25	0.45 ± 0.18	2.32 ± 0.60	5.76 ± 0.66	3.52 ± 0.36
MDA-MB-231	STE	>50 *	>50 *	24.40 ± 7.28	>100 *	>100 *	>100 *
	OTA	31.19 ± 3.41	9.38 ± 2.27	5.13 ± 1.27	>100 *	>100 *	58.92 ± 19.42
	PAT	2.31 ± 0.56	0.61 ± 0.17	0.42 ± 0.13	>25 *	7.30 ± 0.40	2.73 ± 0.21
SH-SY5Y	STE	28.22 ± 11	5.41 ± 1.26	2.91 ± 1.04	>100 *	48.42 ± 7.77	14.81 ± 3.53
	OTA	16.87 ± 5.91	5.80 ± 2.38	2.71 ± 0.87	>100 *	63.24 ± 21.60	5.66 ± 0.36
	PAT	0.45 ± 0.16	0.52 ± 0.22	0.28 ± 0.15	4.93 ± 0.01	3.44 ± 0.56	2.77 ± 0.46

* Highest concentration tested.

**Table 2:** Estimated LD_50_ values based on in vitro IC_50_ values of STE, OTA and PAT in BM-MSC, HUVEC, MDA-MB-231 and SH-SY5Y spheroids. The lowest and highest IC_50_ values shown in Table 1 were selected for each mycotoxin and cell line cultured as spheroids to determine the estimated lowest and highest LD_50_ values.

Mycotoxin	Cell Line	IC_50_ Value (µM)	IC_50_ Value (µg/mL)	log LD_50_ Value (mg/kg)	LD_50_ Value (mg/kg ± SD)
STE	BM-MSCs	100	32.43	2.59	385.53
	HUVECs	100	32.43	2.59	385.53
	MDA-MB-231	100	32.43	2.59	385.53
	SH-SY5Y	100–14.81	32.43–4.80	2.59–2.28	287.49 ± 98.04
OTA	BM-MSCs	100–15.80	40.38–6.38	2.62–2.32	314.44 ± 103.87
	HUVECs	5.14–2.20	2.07–0.89	2.14–2.00	119.9 ± 18.77
	MDA-MB-231	100–58.92	40.38–23.79	2.62–2.54	380.95 ± 37.36
	SH-SY5Y	100–5.66	40.38–2.28	2.62–2.16	281.02 ± 137.29
PAT	BM-MSCs	9.98–8.40	1.54–1.29	2.09–2.06	120.19 ± 3.85
	HUVECs	5.76–2.32	0.89–0.36	2.00–1.86	86.59 ± 14.51
	MDA-MB-231	25–2.73	3.85–0.42	2.24–1.88	125.56 ± 48.98
	SH-SY5Y	4.93–2.77	0.76–0.43	1.98–1.89	86.20 ± 9.21

## Data Availability

Data is contained within the article or supplementary material.

## Supplementary Materials

The following supporting information can be downloaded at: https://www.mdpi.com/article/10.3390/foods13040564/s1. Table S1: Scheme of mycotoxin concentrations used in the study. Figure S1: Morphometric parameters of BM-MSC, HUVEC, SH-SY5Y and MDA-MB-231 spheroids under optimal generation conditions; Figure S2: Representative orthogonal projection of MDA-MB-231 spheroids exposed to PAT 6.25 and 9 µM for 24 h; Figure S3: Representative images of 2D BM-MSCs exposed to PAT 12.5 µM for 24 h; Figure S4: Immunofluorescence staining of E-cadherin and N-cadherin proteins on BM-MSCs spheroids exposed to PAT 100 μM for 72 h.
