# Supplementary material for: Comparative Study of Spheroids (3D) and Monolayer Cultures (2D) for the In Vitro Assessment of Cytotoxicity Induced by the Mycotoxins Sterigmatocystin, Ochratoxin A and Patulin
Source: Foods. 2024 Feb 13;13(4):564. doi: 10.3390/foods13040564 (PMC10887621; doi:10.3390/foods13040564)
Supplement: Supplementary file 1 [file foods-13-00564-s001.zip › foods-2819301-supplementary.pdf]

Table S1. Scheme of mycotoxins concentrations used in the study.

Cell line	Mycotoxin	Range of concentrations tested	
		**2D**	**3D**
BM-MSCs	STE	1.56 – 50 μM	6.25 – 100 μM
	OTA	1.56 – 50 μM	6.25 – 100 μM
	PAT	1.56 – 50 μM	6.25 – 100 μM
HUVECs	STE	1.56 – 50 μM	6.25 – 100 μM
	OTA	1.56 – 50 μM	6.25 – 100 μM
	PAT	1.56 – 50 μM	6.25 – 100 μM
MDA-MB-231	STE	1.56 – 50 μM	6.25 – 100 μM
	OTA	1.56 – 50 μM	6.25 – 100 μM
	PAT	0.28 – 9 μM	6.25 – 25 μM
SH-SY5Y	STE	1.56 – 50 μM	6.25 – 100 μM
	OTA	1.56 – 50 μM	6.25 – 100 μM
	PAT	0.035 – 1.12 μM	3.12 – 12.5 μM

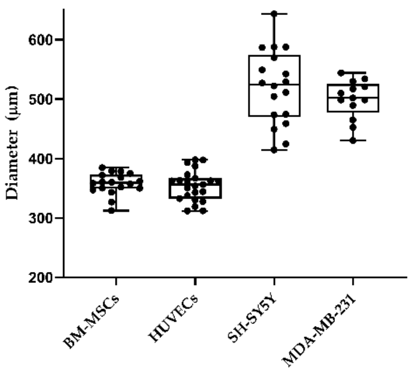

(**a**)

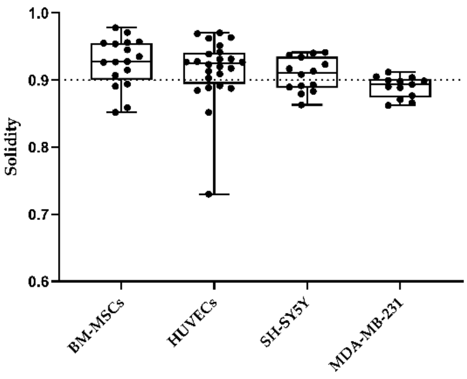

(**b**)

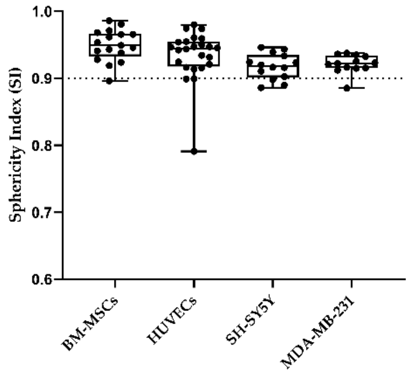

(**c**)

Figure S1. Morphometric parameters of BM-MSCs, HUVECs, SH-SY5Y and MDA-MB-231 spheroids under optimal generation conditions. (**a**) Diameter, (**b**) Solidity and (**c**) Sphericity Index (SI) were obtained from bright-field microscopy images and AnaSP analysis of spheroids at day 1 after seeding for BM-MSCs and HUVECs and at day 7 and 4 after seeding for SH-SY5Y and MDA-MB-231 cells, respectively (N = 3, n = 6). Each point represents a single spheroid, while bars represent the deviation from the minimum to the maximum value of each data set.

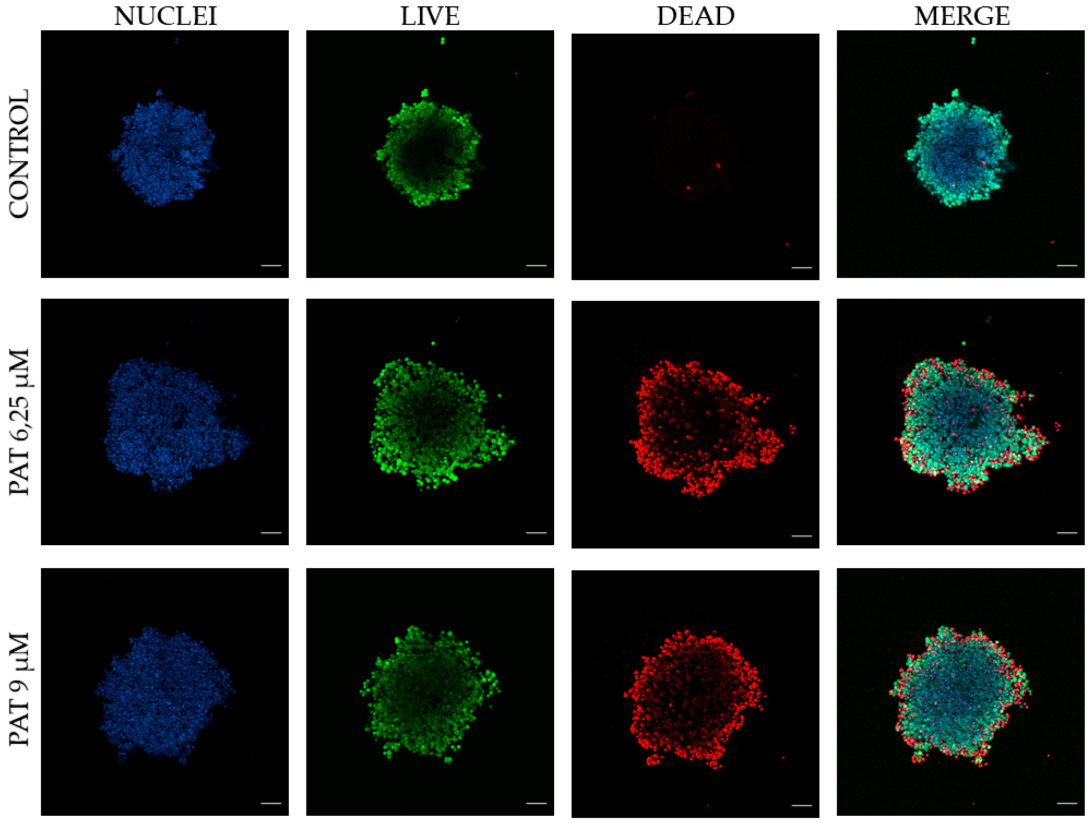

Figure S2. Representative orthogonal projection of MDA-MB-231 spheroid exposed to PAT 6.25 and 9 µM for 24 h. Spheroid viability was evaluated using the LIVE/DEAD™ Viability/Cytotoxicity Assay (Invitrogen®, L3224) on no-fixed cells. The assay allows to simultaneously discriminate live (green) and dead (red) cells using two different probes, the Calcein-AM and Ethidium homodimer-1. The first one is a non-fluorescent cell-permeant that can be converted into a fluorescent molecule (green fluorescence 495-515 nm) by the enzymatic activity of esterases present in viable cells, while the latter is a nucleic acid intercalating agent that is internalised by cells with damaged membranes, enhancing his fluorescence signal (red fluorescence 495-635 nm) after DNA binding. 1.5 mg/ml Hoechst staining solution in PBS was used to stain the nuclei. Spheroids exposed to the same amount of solvent (MeOH) were used as control. Spheroids were observed and imaged under a confocal fluorescence microscope (ZEISS LSM 800 Airyscan, Zeiss Microscopy, Germany). For each spheroid, a z-stack of 10 slices was acquired to capture the information derived from the three-dimensionality of the structure. After acquisition, the images were processed using the orthogonal projection method. Scale bar = 100 µm (objective 10×).

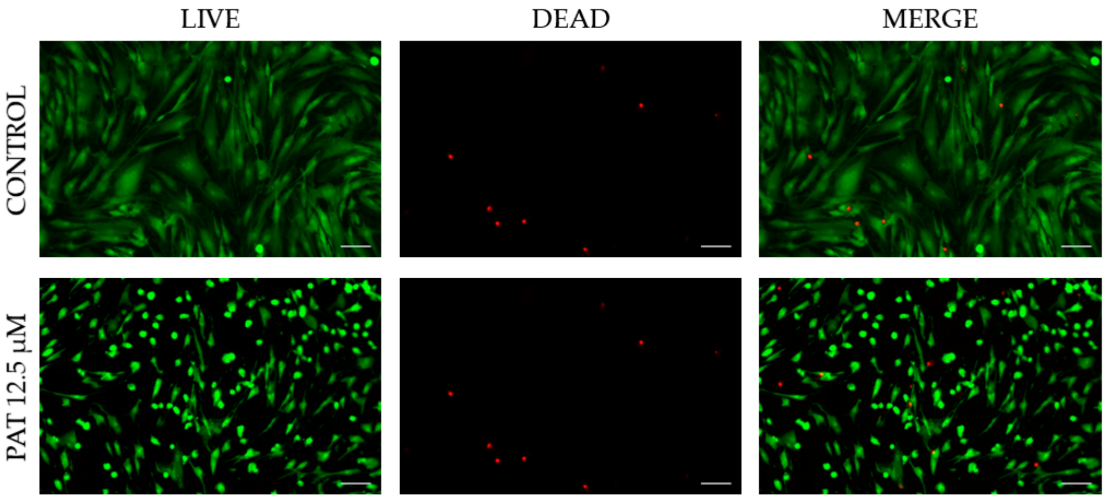

Figure S3. Representative images of 2D BM-MSCs exposed to PAT 12.5 µM for 24 h. Cell viability was evaluated using the LIVE/DEAD™ Viability/Cytotoxicity Assay (Invitrogen®, L3224) on no-fixed cells (see Figure S2 for detailed assay's information). Cells exposed to the same amount of solvent (MeOH) were used as control. Cells were observed and imaged under the inverted fluorescence microscope Zeiss Axio Observer (Zeiss Microscopy, Germany). Scale bar = 100 µm (objective 10×)

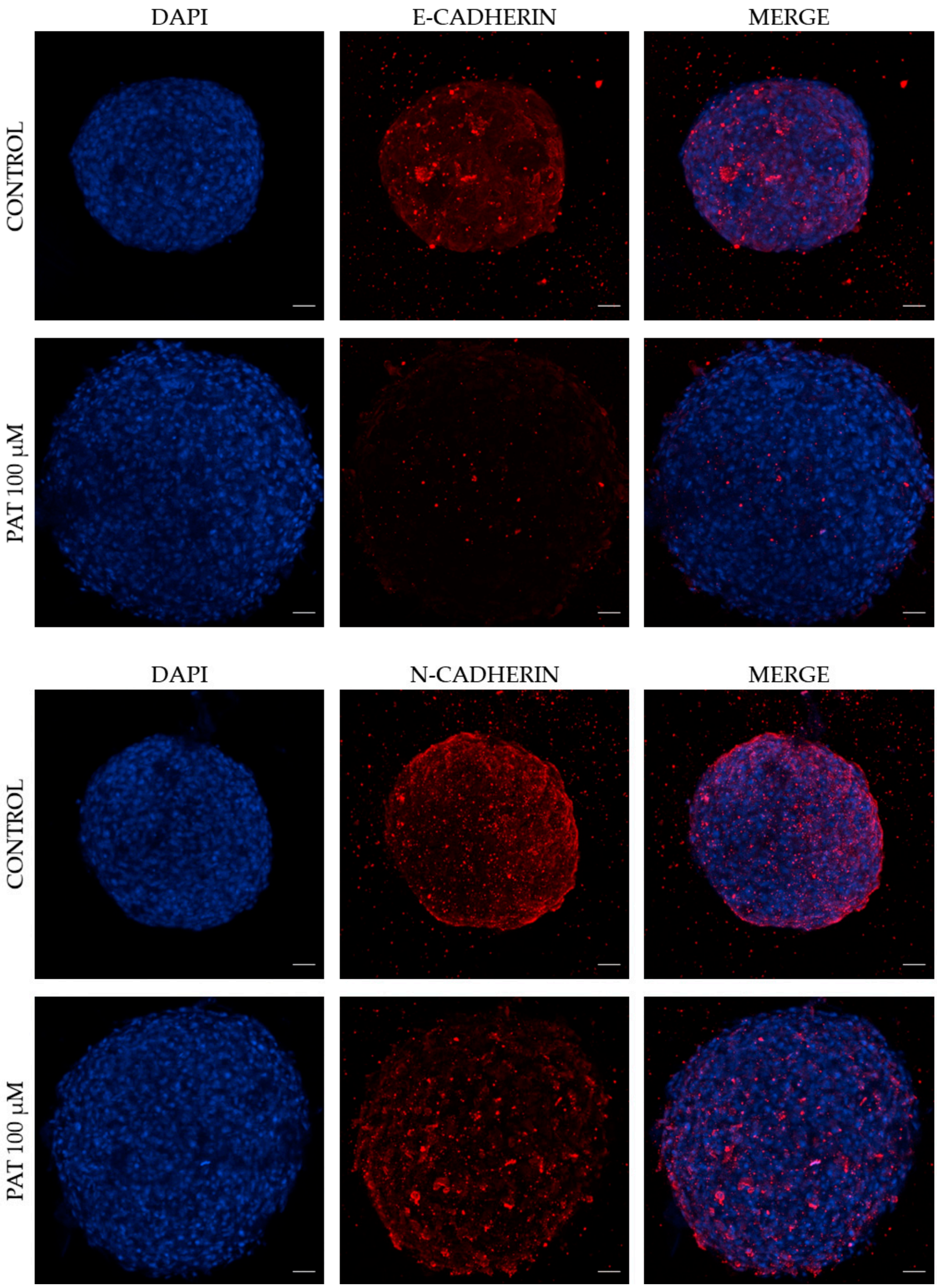

Figure S4. Immunofluorescence staining of E-cadherin and N-cadherin proteins on BM-MSCs spheroids exposed to PAT 100 μM for 72 h. Spheroids were fixed and stained with DAPI to visualize nuclei (blue) and anti-E-cadherin o anti-N-cadherin antibody (red). Spheroids exposed to the same amount of solvent (MeOH) were used as control. Images were obtained by using the confocal fluorescence microscope Zeiss LSM 800 (Zeiss Microscopy, Germany). Scale bar: 50 μm (objective 20x).
